# Clinical-Epidemiological Profile of Patients With Chronic Obstructive Pulmonary Disease Treated at the Pneumology Outpatient Clinic of a Brazilian University Hospital

**DOI:** 10.7759/cureus.75451

**Published:** 2024-12-10

**Authors:** Marcelo V Sarno Filho, Laura N Soares, Margarida C Lima Costa Neves

**Affiliations:** 1 Internal Medicine, Hospital das Clínicas da Universidade de São Paulo, São Paulo, BRA; 2 Medicine, Hospital Universitário Professor Edgard Santos, São Paulo, BRA; 3 Internal Medicine, Departamento de Medicina Interna e Apoio Diagnóstico da Faculdade de Medicina da Bahia da Universidade Federal da Bahia, Salvador, BRA

**Keywords:** chronic obstructive, clinical profile, comorbidity, epidemiology, pulmonary disease

## Abstract

Background

Chronic obstructive pulmonary disease (COPD) is a significant illness that affects many Brazilians. It is a complex and extremely prevalent disease, and thus, understanding the clinical-epidemiological profile of the patients afflicted with this disease is of utmost importance for the adequate management of these patients by multidisciplinary teams.

Objective

The aim of the study was to describe the clinical and epidemiological profile of COPD patients in a specialized outpatient clinic.

Methods

This was a cross-sectional study. The sample comprised 198 patients who attended a specialized outpatient clinic in 2018. All variables were collected from the patients’ medical records.

Results

The mean age of the patients was 69.56 ± 8.98 years (CI 95%: 68.30 - 70.82). Of the 198 patients, 115 (58.1%) were male, while 83 (41.9%) were female. Of all patients, 158 (79.8%) were active smokers or former smokers. The mean value for the forced expiratory volume in the first second (FEV1) was 53.35% ± 21.22 of the expected value (CI 95%: 49.76 - 56.94). The mean number of comorbidities presented by the patients was 2.51 ± 1.81 (CI 95%: 2.25 - 2.76) and the average number of drugs the patients were taking for COPD was 2.83 ± 1.24 drugs (IC95%: 2.66 - 3.01).

Conclusion

This study reveals a complex population, with moderate to severe COPD and a high burden of comorbidities. Thereby, it becomes clear that pulmonologists must consider the COPD patient as a whole due to the high prevalence of factors that can worsen the prognosis in this population.

## Introduction

Chronic Obstructive Pulmonary Disease (COPD) is a prevalent clinical condition marked by chronic airflow obstruction in the lungs. The term "COPD" serves as an umbrella term, traditionally encompassing two key morphological and/or phenotypic manifestations: small airways disease, known as chronic bronchitis, and parenchymal destruction, known as emphysema [1]. These distinct pathological processes can coexist in a single patient, although one is usually more dominant than the other. 

COPD symptoms often follow a chronic and progressive trajectory, with common manifestations, including dyspnea (typically non-wheezy and triggered by physical exertion), dry or productive cough with mucus, fatigue, weight loss, anorexia, and recurrent respiratory infections [1]. Despite the slow and progressive nature of the disease, patients may experience intermittent exacerbations, usually prompted by factors such as viral or bacterial respiratory infections. These episodes, referred to as "COPD exacerbations," are clinically significant due to their association with the further progression of the disease, which can negatively affect patient prognosis [1,2].

Epidemiologically, COPD predominantly affects smokers and individuals over 40 years of age, though it can occur in others as well. The global prevalence was estimated at 11.7% in 2010 [3]. COPD is a leading cause of mortality worldwide, accounting for 3.2 million deaths in 2015 alone [4,5]. In Brazil, the situation mirrors global trends: the PLATINO study (Latin American Project for Pulmonary Obstruction Investigation), published in 2005, found that 15.8% of individuals over 40 in São Paulo had COPD [6].

In addition to pulmonary involvement, many COPD patients also develop comorbidities that exacerbate the clinical progression of the disease [7]. Cardiovascular diseases and diabetes mellitus, for instance, not only increase the risk of disease exacerbations but are also linked to higher mortality rates in COPD patients [7,8]. Other significant comorbidities, such as osteoporosis, anemia, and cerebrovascular events, can also contribute to poorer clinical outcomes [7,8].

A comprehensive understanding of the clinical characteristics of COPD patients is crucial for effective management by a multidisciplinary team. This approach facilitates individualized therapy and helps prevent disease exacerbations. However, in daily clinical practice, a more holistic assessment of these conditions often remains overlooked.

Although the literature emphasizes the importance of studying comorbidities in COPD, especially for disease management and control, there is a lack of data regarding the characterization of COPD patients at the pneumology reference outpatient clinic of the Prof. Edgard Santos University Hospital at the Federal University of Bahia. This study aims to fill this gap by providing insights into the health status of patients attending the clinic, which could inform strategies for better disease control and exacerbation prevention in this population.

## Materials and methods

This was a cross-sectional study conducted with a sample of COPD patients who attended the pneumology outpatient clinic of Professor Edgard Santos University Hospital Complex. Due to operational limitations, a convenience sampling method was used to select participants. The final sample comprised 198 patients who were seen between January 1, 2018, and December 31, 2018, all of whom had a confirmed diagnosis of COPD documented in their medical records.

The following variables were analyzed in the study: sex, ethnicity, age, place of birth, place of origin, prior exposure to biomass smoke, smoking status (current or former smoker), smoking load, presence of comorbidities (as recorded in medical records), peripheral oxygen saturation (SpO2), forced expiratory volume in the first second (FEV1), forced vital capacity (FVC), FEV1/FVC ratio, medications used for COPD management, and evaluation of pulmonary artery systolic pressure (PASP) through transthoracic echocardiography.

Patients were classified based on their FEV1 values into either mild/moderate or severe/very severe airway obstruction groups, in accordance with the guidelines set by the Global Initiative for Chronic Obstructive Lung Disease (GOLD) [1].

Data for all variables were extracted from the hospital's electronic medical record system. Inclusion criteria required patients to have a recorded COPD diagnosis in their medical records and to be 18 years of age or older at the time of data collection. Patients were excluded if they lacked a confirmed COPD diagnosis in their medical records or if their records were incomplete with regard to most of the study variables.

Variables were categorized as either categorical or continuous. Categorical variables were presented as absolute frequencies, relative frequencies, and cumulative relative frequencies, while continuous variables were presented as means or medians, along with standard deviations (±SD). For comparing means and proportions, a 95% confidence interval (95% CI) was used. Comparisons of means were performed using the Student's t-test or the Mann-Whitney U test, and comparisons of proportions were made using the Chi-square test. A p-value of <0.05 was considered statistically significant. The analysis of associations was conducted using the prevalence ratio.

This study was reviewed and approved by the Research Ethics Committee of Professor Edgard Santos University Hospital under opinion number 3.720.252.

## Results

A total of 198 patients were included in this study. The mean age was 69.56 ± 8.98 years (95% CI: 68.30 - 70.82). Among these patients, 115 (58.1%) were male (95% CI: 51.2% - 65%), and 83 (41.9%) were female (95% CI: 35.0% - 48.8%). The mean age for males was 70.70 ± 9.2 years, while for females, it was 67.98 ± 8.4 years (p = 0.035). In terms of ethnicity, 24 patients (12.1%) identified as White, 40 (20.2%) as Black, 132 (66.6%) as Mixed-race, and two (1.0%) did not report their ethnicity. Regarding the place of origin, 77 patients (38.8%) were from Salvador-BA and its metropolitan region, while 103 (52.0%) were from the countryside of Bahia, 16 (8.0%) were from other states in Brazil, one (0.5%) was from another country, and one (0.5%) did not report their place of birth. Regarding residency, 165 patients (83.3%) lived in Salvador-BA and its metropolitan area, while 33 (16.7%) resided in the countryside of Bahia (Table 1).

**Table 1:** Sample characteristics of patients.

Characteristics	Total Sample (n=198)
Age, years	69.56 ± 8.98
Male	115 (58.1%)
Female	83 (41.9%)
Ethnicity	
White	24 (12.1%)
Black	40 (20.2%)
Mixed-race	132 (66.6%)
Did not inform	2 (1.0%)
Place of Birth	
Salvador-BA and metropolitan area	77 (38.8%)
Countryside of Bahia	103 (52.0%)
Other states of Brazil	16 (8.0%)
Other country	1 (0.5%)
Did not inform	1 (0.5%)
Residence	
Reside in Salvador-BA and metropolitan area	165 (83.3%)
Reside in countryside of Bahia	33 (16.7%)

Of the patients evaluated, 158 (79.8%) were current or former smokers, with 93 (58.8%) of them being male (p=0.66). In the smoking and former smoking group, the average smoking history, expressed in pack-years, was 46.12 ± 29.11 (95% CI: 40.88 - 51.36). The average pack-years for men and women were 48.89 ± 29.37 and 42.57 ± 28.65, respectively (p=0.13).

Among the patients with a history of smoking, 135 (85.4%) reported being abstinent from cigarettes. The duration of abstinence was reported by 109 (80.7%) of these patients, with a mean abstinence period of 14.96 ± 12.75 years (95% CI: 12.54 - 17.38). Of the total number of individuals included in the study, 21 (10.6%) had a history of biomass smoke exposure recorded in their medical records (95% CI: 6.3% - 14.9%), while 177 (89.4%) did not (95% CI: 85.1% - 93.7%).

Table 2 presents the characteristics of the study sample according to the severity of airway obstruction.

**Table 2:** Prevalence ration of severe/very severe airflow obstruction according to various comorbidities. PR = Prevalence ratio; 95% CI = 95% Confidence interval

Comorbidity	PR	95% CI
Hypertension	1.12	0.79 – 1.60
Diabetes mellitus	1.24	0.85 – 1.80
Osteoporosis	1.15	0.65 – 2.02
Alcohol Drinking Habit	0.51	0.09 – 2.82
Dyslipidemia	1.05	0.68 – 1.61
Overweight or Obesity	0.88	0.47 – 1.65
Past or active tuberculosis	1.55	1.10 – 2.19

A total of 190 patients (95.9%) had resting peripheral oxygen saturation (SpO2) values recorded in their medical records, measured during consultations. The average SpO2 value was 95.5% ± 3.70% (95% CI: 95.00% - 96.06%). Of these, 183 patients (96.3%) had SpO2 levels of 90.0% or higher.

Regarding transthoracic echocardiography, 119 patients (60.1%) had a record of having undergone the procedure, while 79 patients (39.9%) did not have this exam documented. Among the total sample, 71 patients (35.8%) had a recorded pulmonary artery systolic pressure (PASP) measurement, with an average PASP value of 41.70 ± 16.29 mmHg (95% CI: 37.85 - 45.56).

In the analysis of comorbidities, 182 patients (91.9%) had one or more comorbidities, with an average of 2.51 ± 1.81 comorbidities per patient (95% CI: 2.25 - 2.76). The most commonly observed diseases in the sample and their respective frequencies are detailed in Table 3. The distribution of multiple comorbidities is presented in Table 4.

**Table 3:** Sample characteristics stratified by airflow obstructitiona Variables expressed in n (%) or average ± Standard deviation. * Student's t-test, U of Mann-Whitney, chi-squared, or Fisher’s exact test. COPD: Chronic obstructive pulmonary disease

Characteristics	Mild/Moderate (n=71)	Severe/Very severe (n=66)	p*
Sex			
Male	39 (54.9)	37 (56.1)	0.894
Female	32 (45.1)	29 (43.9)	
Age, years	67.93 ± 8.92	69.85 ± 8.56	0.202
Smoking			
Yes	60 (84.5)	50 (75.8)	0.198
No	11 (15.5)	16 (24.2)	
Number of packs.years	46.34 ± 31.90	49.39 ± 30.29	0.299
Pulmonary Artery Systolic Pressure	37.23 ± 12.02	42.63 ± 11.51	0.127
Comorbidities			
Hypertension			
Yes	37 (52.1)	38 (57.6)	0.521
No	34 (54.9)	28 (42.4)	
Diabetes Mellitus			
Yes	13 (18.3)	17 (25.8)	0.292
No	58 (81.7)	49 (74.2)	
Osteoporosis			
Yes	5 (7.1)	6 (9.1)	0.659
No	66 (92.9)	60 (90.9)	
Alcohol Drinking habit			
Yes	3 (4.2)	1 (1.5)	0.620
No	68 (95.8)	65 (98.5)	
Dyslipidemia			
Yes	13 (18.3)	13 (19.7)	0.836
No	58 (81.7)	53 (80.3)	
Overweight or Obesity			
Yes	8 (11.3)	6 (9.1)	0.674
No	63 (88.7)	60 (90.9)	
Past or active tuberculosis			
Yes	8 (11.3)	17 (25.8)	0.028
No	63 (88.7)	49 (74.2)	
Number of comorbidities	2.34 ± 1.49	2.74 ± 2.22	0.413
Number of COPD drugs	2.55 ± 1.14	3.26 ± 1.22	0.001

**Table 4:** Patients with multiple comorbidities

Number of comorbidities	Number of patients	Relative frequency	Cumulative frequency
0 to 1	62	31%	31%
2 to 3	91	46%	77%
4 or more	45	23%	100%
Total	198	100%	100%

Regarding specific pharmacological therapy for COPD, 193 patients (97.5%) were using at least one medication, as recorded in their medical records (Table 5). The average number of drugs used by patients was 2.83 ± 1.24 (95% CI: 2.66 - 3.01). The types of drugs used and their frequencies are detailed in Table 6. The distribution of multiple drug use in the sample is presented in Table 7. Additionally, Table 6 includes the association analyses between comorbidities and severe/very severe airway obstruction, based on the prevalence ratio.

**Table 5:** Common comorbidities and their simple frequencies

Comorbidities	Number of patients	Relative frequency
Hypertension	113	57.1%
Diabetes mellitus	46	23.2%
Gastroesophageal reflux disease	32	16.2%
Dyslipidemia	33	16.7%
Past or active tuberculosis	30	15.2%
Allergic Rhinitis	26	13.1%
Heart Failure (Non-chagas-related)	18	9.1%
Overweight and Obesity	17	8.6%
Coronary Artery disease	17	8.6%
Cerebrovascular disease	14	7.1%
Lower extremity chronic venous disease	12	6.1%
Osteoporosis	11	5.6%
Depression	10	5.1%
Pulmonary hypertension	11	5.6%
Arrythmia	9	4.5%
Peripheral arterial disease	6	3%
Alcohol Drinking Habit	6	3%
Arthrosis	6	3%
Prostatic hyperplasia	6	3%
Chronic Kidney Disease	4	2%
Glaucoma	4	2%
Prostate cancer	3	1.5%
Anemia	2	1%

**Table 6:** Multiple drugs usage per patient COPD: Chronic obstructive pulmonary disease

Number of COPD specific drugs	Number of patients	Relative frequency	Cumulative frequency
0 to 1	29	14.65%	14.65%
2 to 3	120	60.60%	75.25%
4 or more	49	24.75%	100%
Total	198	100%	100%

**Table 7:** COPD-specific drugs. LABA: long-acting β2 agonist; ICS: inhaled corticosteroid; SABA: short-acting β2 agonist; LAMA: long-acting muscarinic antagonist; SAMA: short-acting muscarinic antagonist; COPD: Chronic obstructive pulmonary disease Drugs described with the symbol “+” were used in combination.

Drug	Number of patientes	Relative frequency of drug usage
LABA + ICS	158	79.8%
SABA	114	57.6%
LAMA	49	24.7%
LABA + LAMA	11	5.6%
ICS	10	5.0%
LABA	8	4.0%
SABA + SAMA	5	2.5%
Phosphodiesterase 4 inhibitors	1	0.5%
Xantines	1	0.5%
Home oxygen therapy	23	11.6%

## Discussion

In this study, the majority of patients were male, a finding consistent with other studies that report a higher prevalence of COPD in men [3,9]. This can be attributed to the higher prevalence of smoking in men, which increases their risk of developing the disease.

Gender differences in COPD have been widely debated. Some studies suggest that women may be more susceptible to the harmful effects of smoking, potentially experiencing more severe disease when exposed to tobacco smoke [10]. A 2010 study [11] found a higher degree of airway obstruction and more severe symptoms among active female smokers with COPD. However, in this study, no significant differences were found between sexes in terms of active or former smoking or smoking load, which may reflect the increasing trend of smoking among women. Moreover, the degree of airway obstruction (according to GOLD [1] criteria) did not show statistically significant differences between males and females.

While smoking is a major risk factor for COPD, some patients develop the disease without a history of tobacco use, likely due to other risk factors such as exposure to biomass smoke, alpha-1-antitrypsin deficiency, and recurrent respiratory infections [12,13]. The literature indicates that 20% to 30% of COPD patients are non-smokers [14], and our study found a 20% prevalence of non-smokers, which is consistent with this expected range.

Biomass smoke exposure is another key risk factor for COPD. A 2003 study [15] conducted with COPD patients at a specialized service in São Paulo found a prevalence of 25.7% of biomass smoke exposure. However, our study found only 10.6% of patients with such exposure. This difference may be due to the fact that most patients were from Salvador and its metropolitan area, where access to electricity and higher socioeconomic conditions likely reduce exposure to biomass smoke [16]. Additionally, incomplete filling of medical records or incomplete transfer of data from paper records to electronic records could have contributed to this difference.

Previous studies have reported that most COPD patients exhibit mild airway obstruction [17,18]. However, in our study, the mean FEV1 was between 50% and 80% of the expected value, classifying the sample as having moderate airway obstruction according to the GOLD criteria [1]. Notably, 48.2% of patients with recorded FEV1 values were classified as having severe or very severe obstruction (FEV1 < 50% of the expected), which is higher than what has been reported in previous studies [17,18]. This suggests that the population in our study may have more severe disease, a finding reflected in their drug profiles, which are consistent with the latest therapeutic recommendations for COPD at the time this study was conducted (GOLD 2018) [1,19].

Regarding peripheral oxygen saturation (SpO2), the study found slightly reduced SpO2 levels in this population. However, the literature has pointed out the imprecision of point measurements of SpO2 via pulse oximetry in COPD patients [20], highlighting the need to complement this test with other assessments such as the 6-minute walk test and arterial blood gas analysis.

Pulmonary arterial hypertension (PAH) is an important factor in COPD, often secondary to hypoxic vasoconstriction of pulmonary vessels [7]. Clinically, pulmonary hypertension is considered a significant prognostic marker, particularly in COPD patients dependent on oxygen therapy [21]. However, the true prevalence of PAH in COPD patients remains uncertain and difficult to estimate in the literature [22].

While invasive measurement of pulmonary artery pressure is the definitive diagnostic method for PAH, the estimation of pulmonary systolic artery pressure (PSAP) through echocardiography is commonly used for screening purposes, though it is no longer recommended as the sole diagnostic criterion [22,23]. In our study, patients had a high average PSAP, which may suggest a high prevalence of pulmonary hypertension. However, only a few patients had a definitive diagnosis of PAH recorded in their medical records, which may reflect incomplete documentation or the absence of information from physicians performing the tests. It is also possible that, due to the difficulty of accessing such exams within the Brazilian Public Health System, echocardiography was performed only on more severe patients, leading to an overestimation bias.

Comorbidities are an important prognostic factor in COPD, as they are associated with increased mortality and exacerbations [7,8,24]. Defining the exact prevalence of comorbidities in this patient group was challenging due to study heterogeneity and confounding factors [7,25]. A 2013 study [26] involving 213 COPD patients found that 97.7% had at least one comorbidity, with 53.5% having four or more comorbidities. The most common comorbidities included systemic hypertension, dyslipidemia, atherosclerotic arterial disease, and osteoporosis [26].

In line with this, our study found that nearly all patients had at least one comorbidity, but a few had four or more, suggesting possible underdiagnosis of certain conditions. This is supported by the lower prevalence of osteoporosis and anemia in our sample, which are frequently observed comorbidities in COPD patients [1,10,25-28]. However, incomplete record-keeping could contribute to these findings, reflecting a limitation of our methodology.

Among the comorbidities studied, tuberculosis stands out as a potential risk factor for COPD development [29,30]. The mechanism through which tuberculosis contributes to chronic airflow obstruction remains unclear, but chronic inflammation and the development of bronchiolitis and pulmonary parenchymal destruction are hypothesized [15,30-32]. In our study, tuberculosis was significantly associated with severe/very severe airway obstruction, which is consistent with previous studies that found reduced FEV1 in patients with a history of tuberculosis [32,33]. This suggests that tuberculosis may not only be a risk factor but also a complicating factor that worsens the prognosis in COPD patients. However, due to the limitations of this study's methodology, further research is needed to confirm this relationship and better understand its implications, considering potential confounders such as smoking, age, and disease duration.

Given the high incidence and prevalence of tuberculosis in Brazil, continuous monitoring of latent infection sites is essential, particularly among elderly COPD patients, many of whom are on systemic or inhaled corticosteroid therapy [34]. In this context, it is crucial for health authorities to standardize and modernize latent tuberculosis testing to improve detection and treatment [35].

The main limitations of this study are related to the methodology of medical record review, as incomplete records may have affected the data. Additionally, the absence of symptom and exacerbation assessments limited the comprehensive evaluation of the patients' health status.

## Conclusions

Finally, we can conclude that this study reveals a complex population with moderate to severe COPD and a high frequency of comorbidities, which is generally consistent with other studies and expected aspects for a specialized outpatient clinic in a university hospital. It is, therefore, emphasized that specialist doctors should focus not only on the pulmonary symptoms of their patients but also on understanding that a broad approach to these individuals is essential, as COPD is a complex systemic inflammatory disease with various comorbidities that aggravate its prognosis.
